# Global research trends on the impact of obesity on male infertility: a bibliometric analysis

**DOI:** 10.3389/fnut.2026.1817082

**Published:** 2026-05-11

**Authors:** Jiuyuan Wang, Wenjing Huang, Mengchen Tan, Nanqi Xu, Qinghu He, Guixiang Sun

**Affiliations:** 1 College of Chinese Medicine, Hunan University of Traditional Chinese Medicine, Hunan, China; 2 The First Clinical College of Traditional Chinese Medicine, Hunan University of Traditional Chinese Medicine, Hunan, China; 3 College of Integrated Chinese and Western Medicine, Hunan University of Traditional Chinese Medicine, Hunan, China

**Keywords:** bibliometric analysis, citespace, male infertility, obesity, paternal epigenetic, sperm

## Abstract

**Background:**

The parallel rise in global obesity and the decline in male fertility have driven research into their causal relationship. While obesity is recognized as a systemic metabolic disorder, the mechanisms by which it impairs male reproduction remain complex. Tracking progress in this field can inform future research directions.

**Methods:**

On January 10, 2026, a literature search on the topic of Obesity and Male Infertility was conducted using two databases, including Web of Science and Scopus. Key publication information, including complete records and references, was extracted from both databases. After merging and cleaning the data from both databases, bibliometric analysis was performed using tools such as CiteSpace and VOSviewer.

**Results:**

The analysis shows exponential growth in the field, with 2012 marking a key shift from niche exploration to mainstream inquiry. China leads in publication volume, but the United States holds greater citation impact and network centrality. A multi-polar institutional network centers on Harvard University, the University of Copenhagen, and the University of Adelaide. Fertility and Sterility and *Andrologia* are the most influential journals by impact and volume, respectively. Chavarro and Eisenberg published the most, while Boeri, Montorsi, and Salonia emerged as key collaborators. Current research focuses on four interconnected areas: oxidative stress-driven sperm DNA damage; the gut-testis axis and metabolic immune regulation; paternal epigenetic inheritance and transgenerational effects; and multidimensional intervention approaches.

**Conclusion:**

This study comprehensively maps the global research landscape of obesity and male infertility, tracing the field's evolution and identifying emerging frontiers. These insights point toward new directions for future research and offer a deeper understanding of the field.

## 1 Introduction

Over the past few decades, the prevalence of obesity worldwide has grown exponentially, evolving into a global epidemic. Recent data indicate that overweight and obesity rates continue to rise worldwide, with this surge no longer confined to Western developed nations but rapidly spreading to middle- and low-income regions such as Asia and Africa (1) Concurrently, obesity has been confirmed as a key risk factor for cardiovascular disease, type 2 diabetes, and various cancers (2), imposing a substantial global disease burden. Against this backdrop, human reproductive health is also in crisis. Recent data indicate that over the past 40 years, sperm counts in Western men have declined by 50% to 60%, with no signs of slowing (3). This synchronized epidemiological trend is not coincidental but suggests a potential link between metabolic disorders and reproductive dysfunction.

Existing evidence indicates that obesity impairs male fertility in multiple dimensions. On the one hand, it directly deteriorates the microenvironment for Spermatogenesis by inducing sleep apnea, disrupting the hypothalamic-pituitary-gonadal axis, and elevating scrotal temperature (4). On the other hand, obesity-induced systemic chronic low-grade inflammation and oxidative stress not only disrupt the testicular immune privilege barrier but also impair sperm DNA integrity and alter key signaling pathways, thereby weakening sperm's fertilization capacity (5). More critically, paternal obesity can transmit metabolic risks to offspring through epigenetic reprogramming of Spermatogonial stem cells, creating transgenerational health hazards (6). Therefore, elucidating how adipose tissue dysfunction disrupts Spermatogenesis via endocrine, immune, and epigenetic networks has become a key research focus in modern reproductive medicine.

Against this backdrop, the volume of literature examining the relationship between obesity and sperm quality has witnessed an exponential surge. However, this rapid accumulation of data complicates the synthesis of existing evidence, underscoring the urgent need for systematic, quantitative evaluation. Bibliometric analysis has established itself as a rigorous methodology for navigating such complex scientific landscapes (7). By analyzing citation patterns and clustering thematic networks, this approach not only maps the current state of knowledge but also disentangles emerging hotspots and evolutionary trends, offering vital guidance for both clinical practice and basic research (8). Moreover, this approach has been widely applied in obesity and sperm-related fields, such as the intersection of obesity and psychology (9) and studies on oxidative stress and male fertility (10). However, despite extensive research on various aspects of obesity and sperm, a bibliometric analysis of this field remains lacking. Given that sperm parameters are the cornerstone of male fertility assessment, bridging this gap is critical. Therefore, this study employs multidimensional bibliometric visualization to delineate the domain's intellectual structure objectively. We aim to identify research frontiers, reveal critical gaps, and provide a roadmap for future investigation, thereby supporting evidence-based decision-making in reproductive medicine.

## 2 Methods

### 2.1 Data sources

The data for this study were sourced from two internationally authoritative databases: Web of Science (WoS) and Scopus. Both provide search and analysis tools capable of generating representative data (11). WoS has long been widely used in bibliometric research due to its stringent journal inclusion criteria, comprehensive citation indexing system, and mature analytical capabilities, offering significant advantages in ensuring data quality and academic reliability.

To broaden data coverage and enhance the comprehensiveness of results, this study also incorporates the Scopus database. Scopus offers broader inclusion advantages in terms of journal volume and disciplinary coverage, effectively supplementing potential omissions in WoS (12). By integrating resources from both databases, this study ensures the quality of the literature while further improving sample completeness and representativeness, providing a more robust data foundation for subsequent knowledge graph analysis.

### 2.2 Search strategy

To ensure data timeliness and accuracy, this study conducted literature searches in the Web of Science Core Collection and Scopus databases on January 10, 2026. The search strategy was strictly constructed around the two core themes of “obesity” and “male Sperm,” optimized by referencing high-impact meta-analyses. The specific search terms are as follows:

**WoS:** TS = (obes^*^ OR overweight OR adiposity OR “body mass index” OR BMI) AND TS = (sperm^*^ OR semen OR “semen quality” OR “sperm quality” OR “sperm motility” OR “sperm concentration” OR “sperm morphology” OR “semen parameter^*^” OR “sperm DNA fragmentation” OR “male infertility” OR fertility).

**Scopus:** TITLE-ABS-KEY(“body mass index” OR bmi OR obes^*^ OR overweight OR adiposity) AND TITLE-ABS-KEY(Sperm^*^ OR semen OR “male fertil^*^” OR “male infertil^*^” OR “semen quality” OR “sperm quality” OR “sperm motility” OR “sperm concentration” OR “sperm morphology” OR “sperm DNA fragmentation” OR azoosperima OR oligozoospermia OR asthenozoospermia OR teratoospermia).

**Figure 1:**
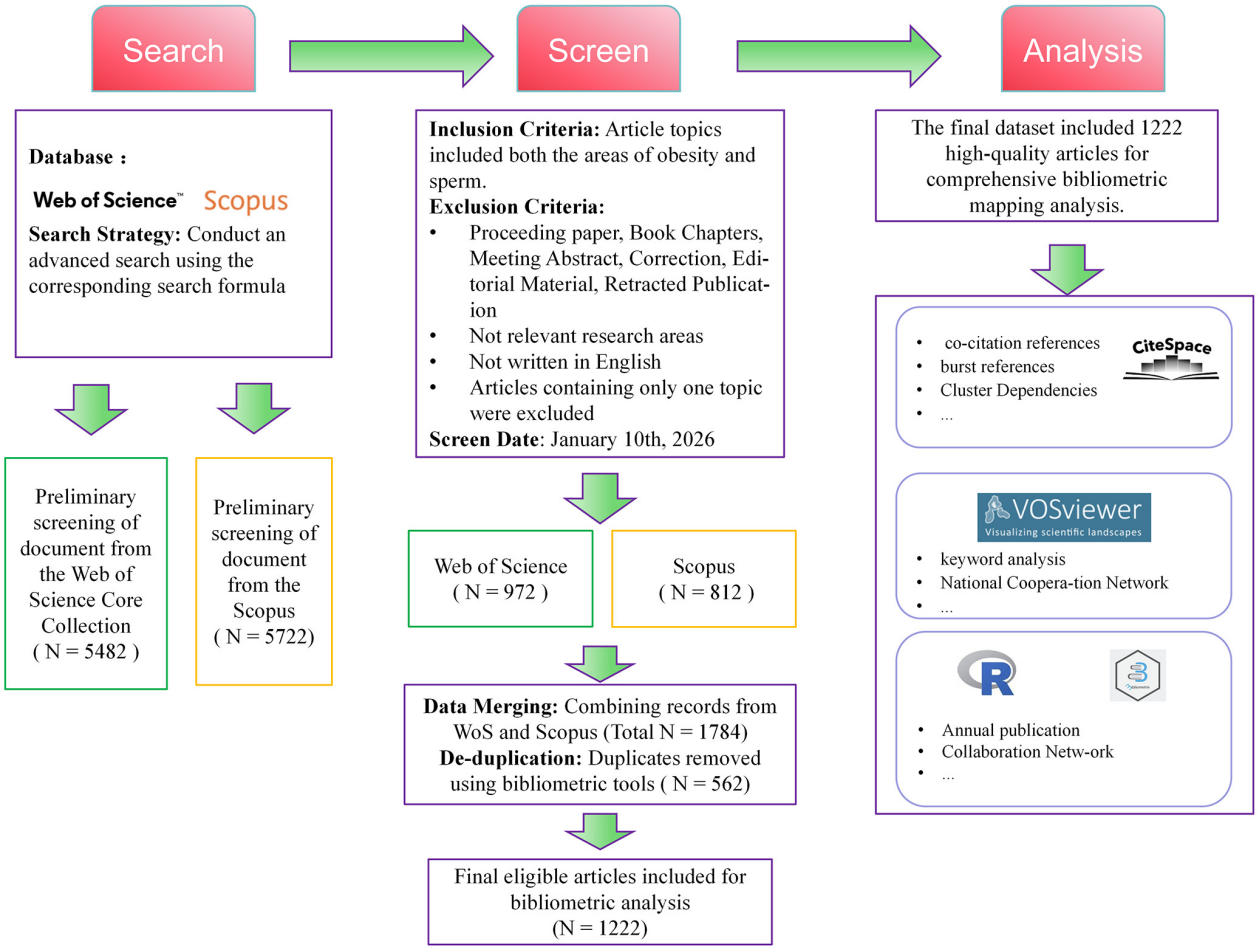
Article screening flow chart.

The literature screening process followed the PRISMA (Preferred Reporting Items for Systematic Reviews and Meta-Analyses) guidelines for systematic reviews, as outlined in Figure 1. First, the document types were restricted to original research and reviews, and the language was limited to English. An initial search in WoS yielded 5,482 documents. After excluding irrelevant topics and non-target languages, 3,599 documents remained. Further screening ultimately resulted in the inclusion of 972 documents. A search in Scopus yielded 5,722 articles. After applying the same criteria, 2,200 articles remained, and 812 were ultimately included.

To minimize selection bias, four independent researchers conducted an initial review of titles and abstracts. In cases of disagreement, the first author made the final decision, and a fifth senior researcher performed final quality control on the selected data. Finally, bibliometric tools were used to merge data from both databases, resulting in the removal of 562 duplicate records. Ultimately, 1,222 high-quality articles were included in the subsequent analysis.

### 2.3 Bibliometric software

This study employs CiteSpace (v6.2.4 R), VOSviewer (v1.6.19), and the R bibliometrix package (v4.1.3) to construct multidimensional knowledge maps. The aim is to comprehensively analyze the evolutionary trajectory of obesity and male sperm research through complementary multi-tool approaches. First, VOSviewer primarily generates clustering views of keyword co-occurrence and collaboration networks, visually revealing the field's foundational knowledge architecture and core communities. Second, CiteSpace focuses on temporal analysis, utilizing its unique emergence detection and timeline views to aid understanding of progress and trends. Additionally, the R bibliometrix package is employed for macro-level descriptive statistics. We also employ the H-index to evaluate the academic influence of scholars, institutions, journals, and countries, while referencing the Impact Factor (IF) from the latest Journal Citation Reports (JCR) as the standard for measuring journal quality.

## 3 Result

### 3.1 Trends in scientific output in the field of obesity/male infertility

#### 3.1.1 Publication volume and citation trends

**Figure 2:**
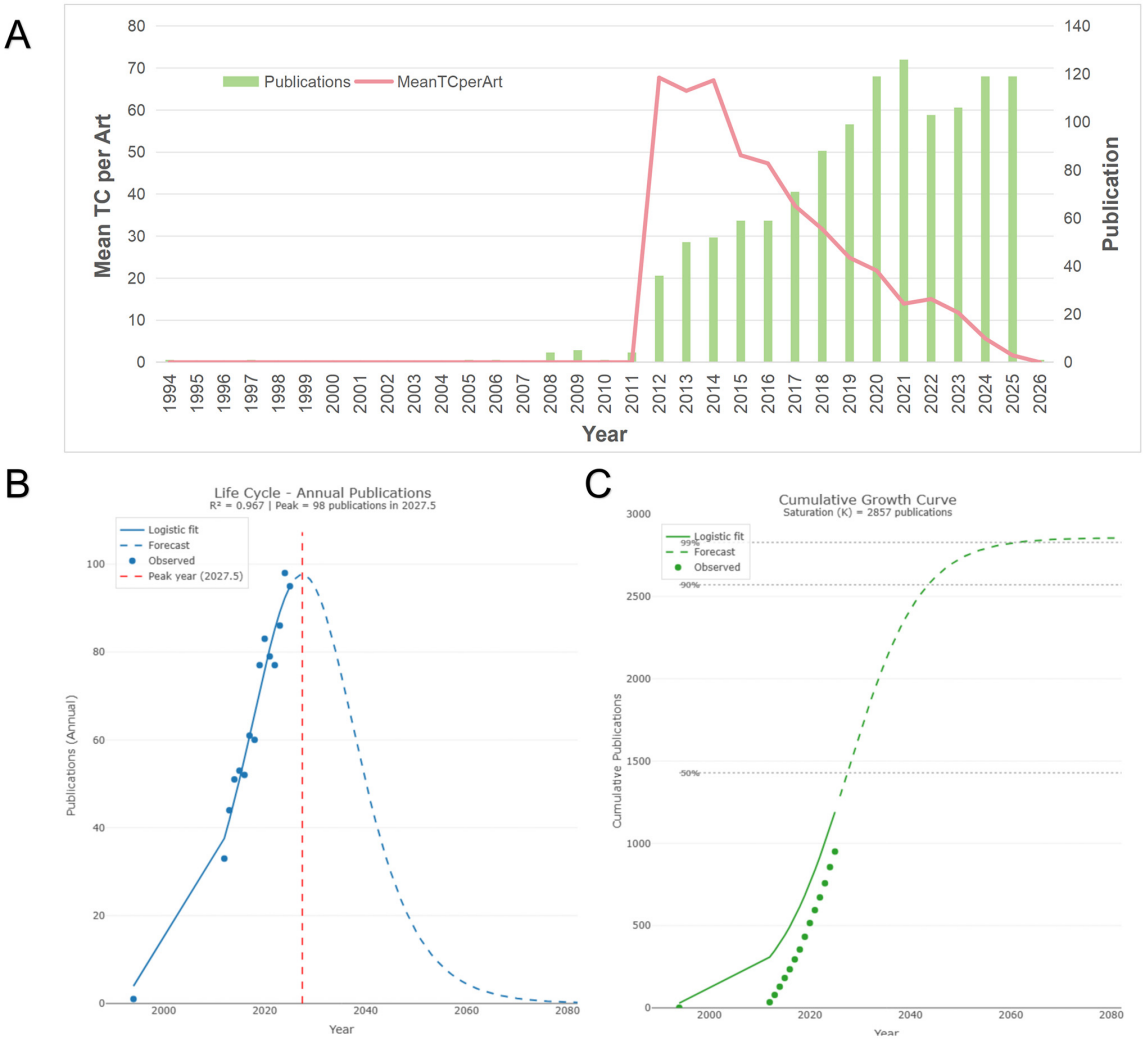
**(A)** Annual publication volume and citation impact. The green bars represent the number of annual publications. The red line indicates the mean total citations per article. **(B)** Life cycle analysis of annual publications. The blue curve represents the logistic growth model fit. **(C)** Cumulative growth curve of publications. The solid green dots represent observed cumulative data, while the dashed line represents the predicted logistic S-curve.

Publication volume over time serves as a powerful indicator of research trends and progress in specific fields. This study included 1,222 papers, with their annual publication volume and average citation count shown in Figure 2A. Publication trends in this field can be divided into three phases. The first phase, spanning 1994–2011, featured persistently low publication volume, with most years producing 0–5 papers. The second phase, spanning 2012–2021, saw publication volume surge from 20 papers in 2012 to 36 in 2013, then sustained growth, reaching a peak of 126 in 2021. The third phase began after 2022, with annual publication volume fluctuating within a higher range, maintaining 119 papers in both 2024 and 2025.

Mean total citations per article (MeanTCperArt) is a key metric for measuring the average academic impact and quality of papers published in a given year. This indicator reflects the extent to which research outcomes published in that year garner attention and citations from the academic community in subsequent years. The most striking feature in Figure 2A is the exceptionally high average citations per paper between 2012 and 2016, with the mean consistently ranging from 47.31 to 67.72. From 2017 to 2020, as the number of publications increased annually, citations inevitably became diluted, leading to a gradual decline in average citations per paper from 37.18 to 21.8. Beginning in 2021, the average number of citations per paper decreased significantly.

#### 3.1.2 Life cycle analysis of scientific output

To investigate the developmental stage and future trends in this field, the Life Cycle feature of Bibliometrix was employed for exploration, as shown in Figures 2B, C. The coefficient of determination, R^2^ = 0.967, indicates that the model explains 96.7% of the variation in publication volume. This demonstrates that the field's development trajectory closely follows a logistic growth pattern, yielding highly credible predictive results.

Figure 2B displays the fitted curve for annual publication volume in this field. The observed data points (blue dots) closely follow the upward slope of the fitted curve, indicating that the field is currently in an late exponential phase of logistic growth. The model predicts that publication volume will peak in 2027.5, with an estimated annual output of approximately 98 papers. This implies that research activity in this field will continue to rise over the next 1–2 years. After 2027, as core scientific questions are progressively resolved, the annual publication volume is expected to decline gradually, entering a mature phase.

Figure 2C displays the cumulative publication volume fitting curve for this field. The current cumulative publication count is in the steep ascent phase of the S-curve and has not yet crossed the inflection point. This further confirms that the field is in a dynamic phase of rapid development, far from reaching saturation. It is projected that around 2027, the cumulative publication count will reach half of its saturation value.

### 3.2 National analysis in the field of obesity/male infertility

A total of 220 countries/regions have published articles in this field. Table 1 lists the top ten countries by publication volume. Among them, China (*n* = 204) and the United States (*n* = 193) ranked first and second, respectively, and were significantly higher than other countries. They were followed by Italy (*n* = 93), Australia (*n* = 57), and Spain (*n* = 49). Regarding citation frequency, the United States leads with 9,139 total citations, followed by China (4,680) and Italy (3,364). Denmark (2,837 citations) and Australia (3,273 citations) demonstrate relatively high citation frequencies despite their lower publication volumes.

**Table 1:** Top 10 countries/regions in terms of publications.

Name	Publication	Times cited	H-index
USA	193	9,139	53
CHINA	204	4,680	34
ITALY	93	3,364	32
AUSTRALIA	57	3,273	29
SPAIN	49	2,056	21
BRAZIL	46	2,201	21
DENMARK	43	2,837	25
ENGLAND	45	1,596	19
FRANCE	41	1,719	17
IRAN	39	834	17

#### 3.2.1 Analysis of global collaborative networks

**Figure 3:**
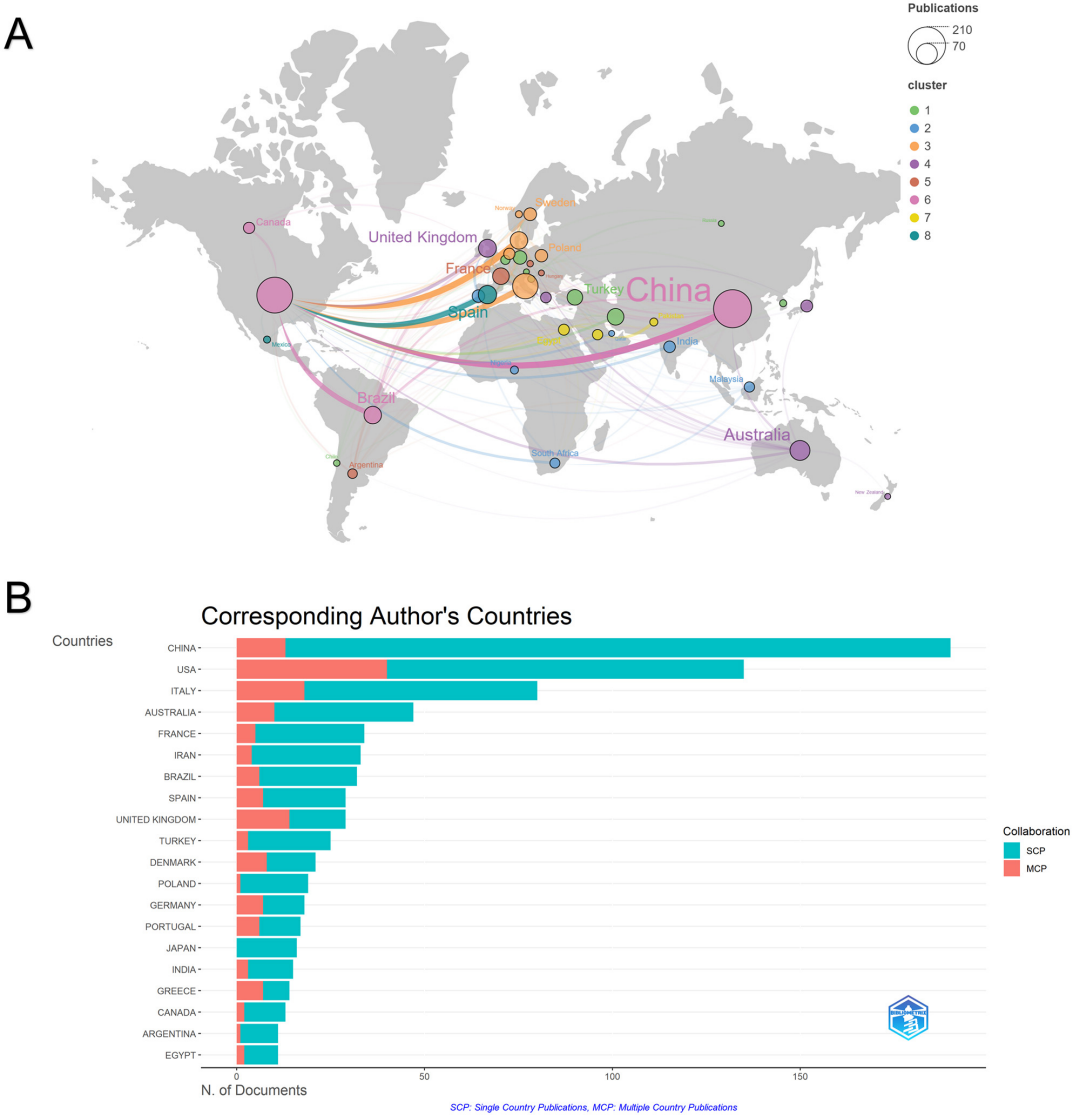
**(A)** The visualization of the global collaboration network. Nodes represent countries/regions, with sizes proportional to the number of publications. Curved lines denote collaborative links between countries, with thickness indicating the strength of cooperation. Colors represent distinct collaboration clusters. **(B)** The top 10 most active funding agencies. MCP denotes Multiple Country Publications, indicating articles with international co-authors; SCP denotes Single Country Publications, representing domestic research. The MCP ratio reflects each country's degree of internationalization.

To further illustrate the geographic distribution and intensity of global scientific collaboration, a network map of country/region partnerships was constructed using VOSviewer software. Figure 3A shows that the United States is the absolute hub of the global scientific collaboration network. Its total link strength (TLS) is 141 (see the Supplementary material), far surpassing that of any other nation. This signifies that the United States not only produces abundant research output but also serves as a pivotal hub connecting global scientific resources. Its outgoing links are the thickest and most extensive, radiating across Europe, Asia, and the Americas, indicating a worldwide network of partners. Simultaneously, global collaboration has formed eight closely connected country clusters. Among these, the pink cluster (including China, the United States, Canada, Brazil, etc.) stands out, while European nations dominate the orange cluster.

The country of the corresponding author typically represents the dominant research force and primary funding source. Figure 3B displays the top 20 countries/regions by publication volume and their international collaboration patterns. Collaboration patterns vary significantly across nations, as evidenced by the MCP% metric. Although China leads in publication volume, its MCP count stands at only 13 papers, resulting in a low international collaboration rate (MCP%) of 6.8%. The vast majority of papers were authored independently by Chinese scholars. The United States maintains a high output while sustaining a strong international collaboration rate. Italy, Australia, and Spain also exhibit relatively healthy collaboration rates.

#### 3.2.2 Analysis of funding support

To further illustrate the distribution and intensity of research funding, the top ten funding agencies supporting this field are presented in Table 2. Research funding in this field primarily originates from a small number of countries or regions with high levels of scientific investment. The National Institutes of Health (NIH) in the United States and the National Natural Science Foundation of China (NSFC) rank first in joint rankings, each supporting 71 related studies. Funding agencies at the European Union level (EU & ERC) rank third, collectively supporting 35 publications. Next were the National Health and Medical Research Council (NHMRC) (*n* = 28) and the United Kingdom National Funding (*n* = 17).

**Table 2:** Top 10 funding agencies in terms of publications.

Funding name	Publications
NIH (National Institutes of Health)	71
China National Funding (NSFC)	71
European Union (EU & ERC)	35
NHMRC (Australia)	28
United Kingdom National Funding (UKRI, MRC, NIHR)	17
Instituto Politécnico Nacional	30
Brazil National Funding (CNPq & CAPES)	16
FUNDACAO PARA A CIENCIA E A TECNOLOGIA FCT	17
Spanish Government	14
Novo Nordisk Foundation	12

### 3.3 Institutional analysis in the field of obesity/male infertility

A total of 3,414 institutions worldwide published papers in this field, with the top ten institutions by publication volume shown in Table 3. Harvard University (*n* = 36) ranked first, followed by INSERM (*n* = 29), the University of Copenhagen (*n* = 27), the University of Adelaide (*n* = 26), and Assistance Publique-Hôpitaux de Paris (*n* = 21), in that order. Among these, the University of Copenhagen and the University of Adelaide both achieved an H-index of 21, ranking highest. The University of Copenhagen recorded the highest citation count (2,252 citations).

**Table 3:** Top 10 research institutions in terms of publications.

Institution	Publication	Times cited	H-index
Harvard University	36	1,729	20
INSERM	29	1,508	15
University Of Copenhagen	27	2,252	21
University Of Adelaide	26	1,947	21
Assistance Publique Hopitaux Paris	21	1,285	11
Ciber Research Network	18	782	13
Stanford University	20	971	10
San Raffaele Vita-Salute University	16	354	12
Cleveland Clinic Foundation	17	1,449	11
University Of Florence	17	1,192	13

**Figure 4:**
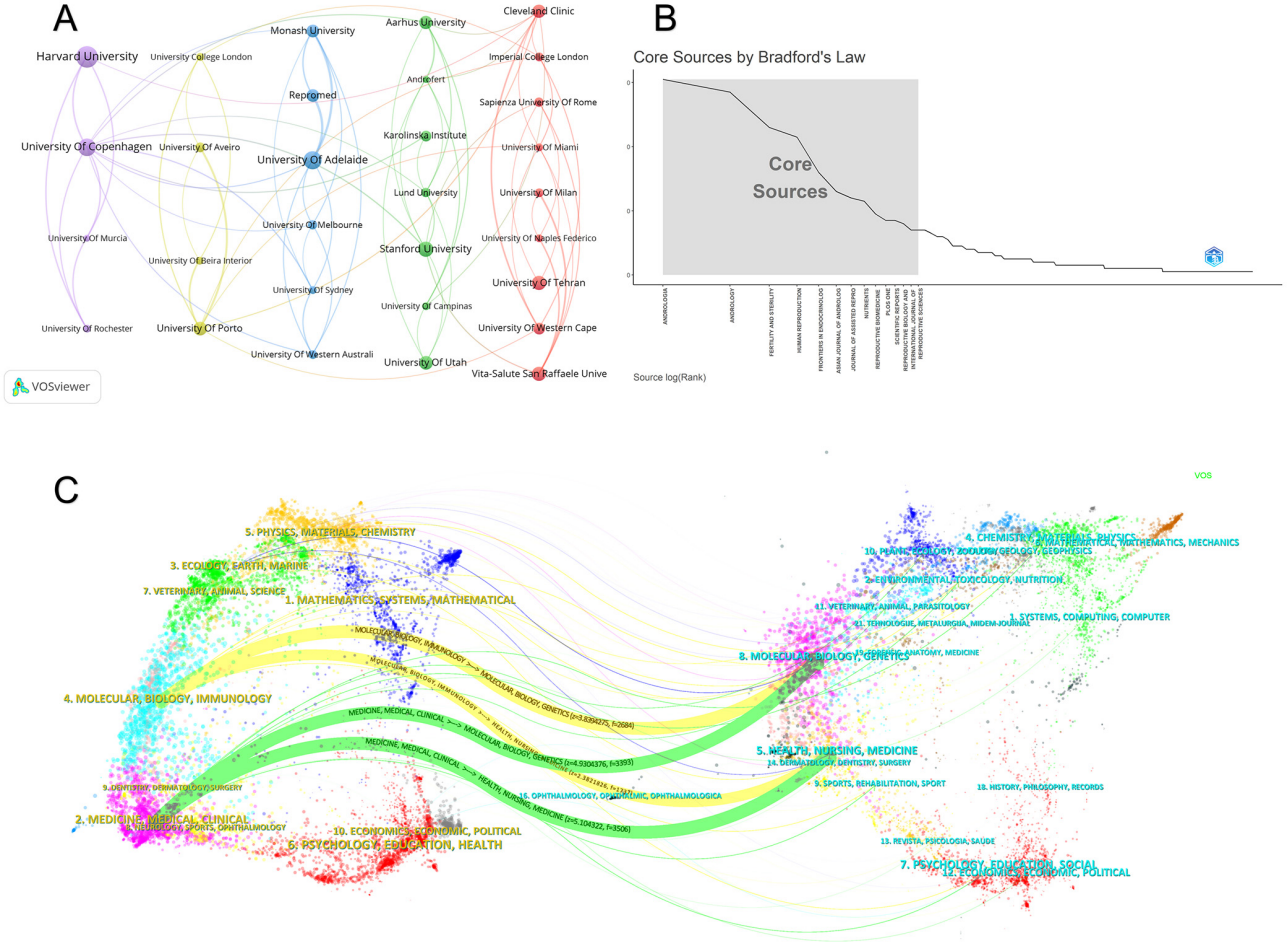
**(A)** Institutional cooperation network. The node's size indicates the number of publications, the line's thickness indicates the strength of the collaboration, and the color indicates clustering. **(B)** Bradford's Law. **(C)** The journal dual-map overlays map. The left side indicates the citing literature, and the right side indicates the cited literature. A line indicates a citation relationship, with the left side pointing to the right side, indicating that the article on the left cites the article on the right.

Institutions serve as the concrete vehicles for implementing research projects, and the configuration of their collaborative networks reflects the aggregation and flow characteristics of academic resources. Using VOSviewer, we constructed a collaborative network map of major research institutions, shown in Figure 4A. These institutions formed four distinct clusters. The blue cluster, centered around the University of Adelaide, includes Monash University, the University of Melbourne, and the University of Sydney, forming a highly cohesive domestic Australian collaboration network. The purple cluster, centered around Harvard University and the University of Copenhagen, represents a collaboration between American universities and Nordic institutions. The green cluster includes Stanford University and the University of Utah from the United States, as well as the Androfert Fertility Center from Brazil. The red cluster comprises institutions such as the Cleveland Clinic, Imperial College London, and Sapienza University of Rome.

The University of Adelaide and the University of Copenhagen serve as core hubs within this collaborative network, both ranking first with a TLS of 29. This indicates that these institutions not only possess robust research capabilities themselves but also actively lead cross-institutional and transnational research collaborations, functioning as pivotal nodes connecting the global academic network. In contrast, Harvard University's TLS of 14 indicates that, while it produces high-quality research, its collaborative breadth or frequency falls short of those of the aforementioned institutions.

### 3.4 Journal analysis in the field of obesity/male infertility

These articles were published across 501 journals. Based on Bradford's analysis of source journals, core journals in this research field were identified, as shown in Figure 4B. Results indicate that publications are concentrated in a small number of high-frequency journals, with ANDROLOGIA and ANDROLOGY ranking first and second, respectively, as the primary sources of literature in this field. The top ten core journals by publication volume are listed in Table 4. ANDROLOGIA (*n* = 54) and ANDROLOGY (*n* = 51) ranked first and second, followed by FERTILITY AND STERILITY (*n* = 46) and HUMAN REPRODUCTION (*n* = 42). Publications in the remaining journals ranged between 17 and 28 articles. In terms of citation frequency, FERTILITY AND STERILITY (2,779 citations) and HUMAN REPRODUCTION (2,433 citations) had the highest total citation counts, followed by ANDROLOGY (1,529 citations) and ANDROLOGIA (1,399 citations). Among the top ten journals by publication volume, most are in the Q1 quartile. Asian Journal of Andrology and PLoS ONE are in the Q2 quartile, while ANDROLOGIA is in the Q3 quartile.

**Table 4:** Top 10 Journals in terms of publications.

Journal	Publication	Times cited	H-index	IF	JCR
ANDROLOGIA	54	1,399	22	2	Q3
ANDROLOGY	51	1,529	25	3.4	Q1
FERTILITY AND STERILITY	46	2,779	30	7	Q1
HUMAN REPRODUCTION	42	2,433	27	6.1	Q1
ASIAN JOURNAL OF ANDROLOGY	28	935	17	2.7	Q2
FRONTIERS IN ENDOCRINOLOGY	25	290	9	4.6	Q1
JOURNAL OF ASSISTED REPRODUCTION AND GENETICS	24	415	11	2.7	Q1
PLOS ONE	17	949	14	2.6	Q2
REPRODUCTIVE BIOMEDICINE ONLINE	17	936	12	3.5	Q1
SCIENTIFIC REPORTS	17	342	11	3.9	Q1

To reveal the interdisciplinary characteristics and knowledge flow trajectories in this field, a dual-map overlay was constructed using CiteSpace software, as shown in Figure 4C. Two primary knowledge flow pathways emerge: the green pathway extends from MEDICINE, MEDICAL, CLINICAL to MOLECULAR, BIOLOGY, GENETICS (z = 4.93, f = 3,393), and from HEALTH, NURSING, MEDICINE to MOLECULAR, BIOLOGY, GENETICS (z = 5.1, f = 3,506). The yellow path extends from MOLECULAR, BIOLOGY, IMMUNOLOGY to MOLECULAR, BIOLOGY, GENETICS (z = 3.83, f = 2,684).

### 3.5 Author analysis in the field of obesity/male infertility

A total of 5,276 authors have published articles in this field. The top ten authors by publication volume are listed in Table 5. Jorge E Chavarro, and Eisenberg Michael L, tied for first place with 16 publications each, followed by Alves Marco G. (*n* = 14), Salonia, Andrea (*n* = 13), McPherson Nicole (*n* = 13), and others. Among them, Chavarro Jorge E leads with 1,218 citations and an H-index of 13. Although Jorgensen, Niels ranks lower in publication volume, his citation count and H-index are both notably high.

**Table 5:** Top 10 authors in terms of publications.

Author	publication	times cited	H-index
Chavarro, Jorge E	16	1,218	13
Eisenberg, Michael L	16	849	9
Alves, Marco G	14	726	11
Salonia, Andrea	13	300	11
Mcpherson, Nicole	13	484	11
Oliveira, Pedro F	13	522	11
Montorsi, Francesco	12	246	9
Boeri, Luca	12	246	9
Jorgensen, Niels	11	953	11
Ventimiglia, Eugenio	11	247	9

#### 3.5.1 Author collaboration network analysis

**Figure 5:**
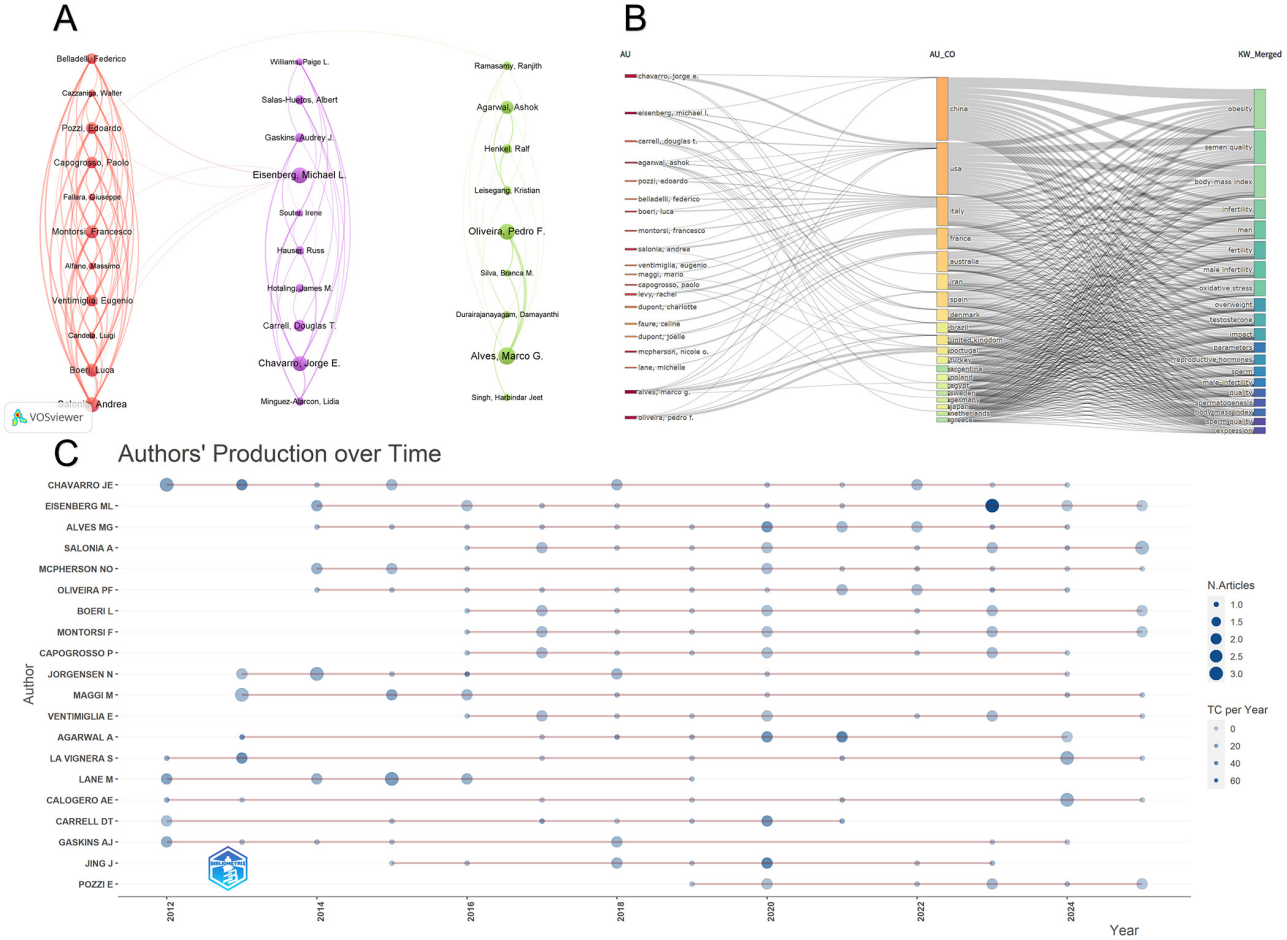
**(A)** Author cooperation network. **(B)** Three-field plot of author-country-keyword relationships. **(C)** Authors' production over time chart. The node's size indicates the number of publications, and the colour's shade indicates the number of citations.

The top 20 author collaboration network, constructed using VOSviewer, reveals multiple stable collaborative networks among authors in this field, as shown in Figure 5A. The author collaboration network exhibits a multi-cluster structure. Within this network, Luca Boeri, Francesco Montorsi, and Andrea Salonia all achieved TLS values exceeding 70, indicating not only multiple collaborative relationships with their co-authors but also a high frequency of collaboration. Eugenio Ventimiglia and Paolo Capogrosso similarly exhibit high TLS values, further confirming their central collaborative positions within the author collaboration network.

#### 3.5.2 Keyword analysis of authors and their countries of affiliation

The three-field author-country-keyword plot constructed with Bibliometrix reveals correspondences among core authors, their countries of affiliation, and research keywords, in Figure 5B. Multiple core authors form stable connections with countries such as the United States, Italy, China, Denmark, and Australia, indicating that these nations host a significant concentration of active researchers in this field.

Distinct connections exist between different countries and multiple keywords. Keywords such as “obesity,” “body mass index,” “semen quality,” “male infertility,” “fertility,” and “oxidative stress” connect to multiple countries, forming the most prominent research theme nodes in the plot. Conversely, specific keywords (e.g., “reproductive hormones,” “Spermatogenesis,” and “testosterone”) are primarily linked through a limited number of countries and authors, indicating relatively concentrated research focus.

#### 3.5.3 Annual publication activity of the top 20 active authors

Figure 5C displays the annual publication and citation activity of the top 20 high-frequency authors. The figure indicates that most authors became active in this field between 2012 and 2016. Jorge E Chavarro has been active since 2012. Although his publication frequency was not continuous year-round, the darker colors of the early circles (2012, 2014) indicate that her early research on obesity and reproductive health was foundational to the field. Agarwal A, a leading expert in andrology, maintains a continuous research trajectory from 2012 to 2024, with particularly dark circles in 2020–2021. This indicates not only high productivity but also exceptionally high citation rates during this period, positioning him as a driving force in advancing the field. Eisenberg ML exhibits a prominent dark blue circle in 2023. This indicates he published an article of immense influence within the field that year. Authors La Vignera S and Calogero Ae both exhibit exceptionally prominent circles in 2024, large and intensely colored. This signifies that these scholars have produced high-quality work in the field of obesity/male infertility, making them among the most active and cutting-edge figures in this research frontier.

### 3.6 Keyword analysis in the field of obesity/male infertility

**Figure 6:**
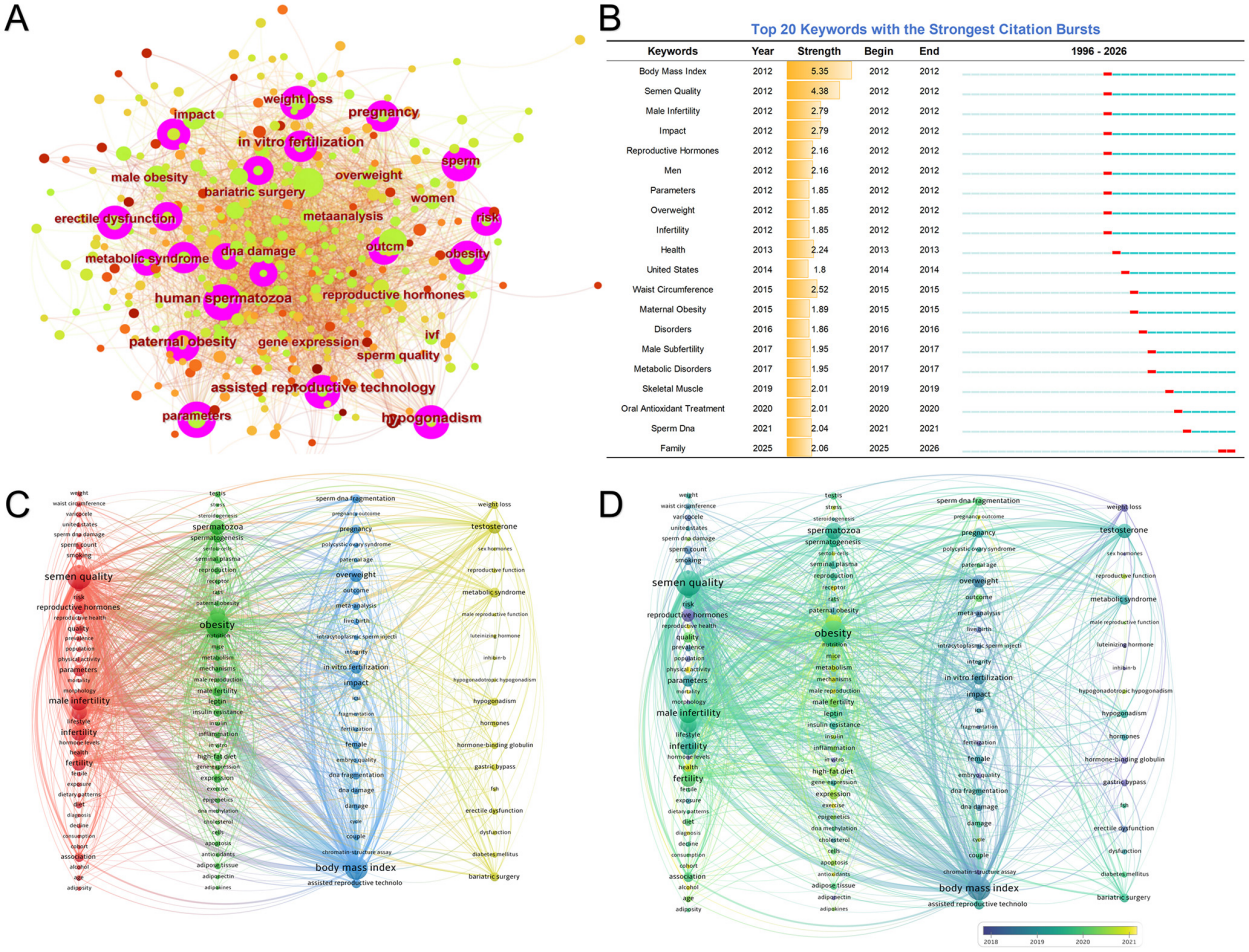
**(A)** Keyword co-occurrence network. Each node represents a keyword, with the node size indicating its frequency of occurrence. Links between nodes represent co-occurrence relationships. **(B)** Top 20 keywords with the strongest citation bursts. The blue line represents the entire study period, and the red segment indicates the duration of each keyword's citation burst. **(C)** Keyword co-occurrence network by VOSViewer. The node size indicates how often the keyword appears; the larger the node size, the higher the frequency. Node color indicates different clusters. Lines indicate that two keywords co-occur in the same paper or papers. **(D)** Keyword temporal Overlay map. Different node colors indicate different years.

This field comprises 1,685 keywords, with the most frequently occurring terms including sperm, paternal obesity, human spermatozoa, hypogonadism, and assisted reproductive technology, as shown in Figure 6A. To identify the evolution of research hotspots and emerging trends in this field, CiteSpace software was used to detect keyword emergence, yielding 20 emergent keywords, as shown in Figure 6B. Body mass index exhibited the highest emergence strength (Strength = 5.35), followed by “semen quality” (Strength = 4.38). Keywords gaining prominence in recent years include “family” and “Sperm DNA.”

#### 3.6.1 Keyword co-occurrence network analysis

To reveal research hotspots and knowledge structures in this field, a keyword co-occurrence network was constructed using VOSviewer (Figure 6C). By setting a minimum occurrence threshold, 120 high-frequency keywords were selected. Based on the strength of keyword connections, the network was divided into four major clusters, each representing distinct research themes.

Cluster 1 (Red Cluster): Core keywords include body mass index, semen quality, male infertility, lifestyle, diet, smoking, waist circumference, etc. This cluster primarily focuses on the epidemiological association between obesity and male infertility, as well as the impact of lifestyle factors.

Cluster 2 (Green Cluster): Core keywords include oxidative stress, spermatozoa, inflammation, adipokines, leptin, epigenetics, and DNA methylation. This theme emphasizes how adipokines secreted by adipose tissue, oxidative stress responses, and epigenetic alterations affect Sperm.

Cluster 3 (Blue Cluster): Core keywords include *in vitro* fertilization, ICSI, pregnancy, live birth, and DNA fragmentation. This cluster primarily examines obesity's impact on assisted reproductive technology success rates, embryo quality, and pregnancy outcomes, while also addressing paternal obesity's effects on offspring health.

Cluster 4 (Yellow Zone): Core keywords include testosterone, metabolic syndrome, bariatric surgery, hypogonadism, and weight loss. This research theme covers male hypogonadism, hormonal characteristics of metabolic syndrome, and the reproductive function-enhancing effects of weight-loss surgery.

#### 3.6.2 Keyword co-occurrence temporal network analysis

Figure 6D illustrates the temporal evolution of keywords. Early research keywords from 2018 and prior focused on fundamental clinical correlations and hormone-level studies, such as inhibin B, FSH, and hormone levels. By 2018–2019, keywords gradually shifted toward specific pathological states, including metabolic syndrome and DNA fragmentation. After 2020, a new cohort of keywords emerged representing the latest research frontiers. These include studies deepening understanding of mechanisms (epigenetics/inflammation), novel biomarkers and interventions (adipokines, sperm DNA damage, receptor), and specific populations and health perspectives (reproductive health, cohort).

### 3.7 Reference analysis in the field of obesity/male infertility

#### 3.7.1 Co-citation network of references

**Figure 7:**
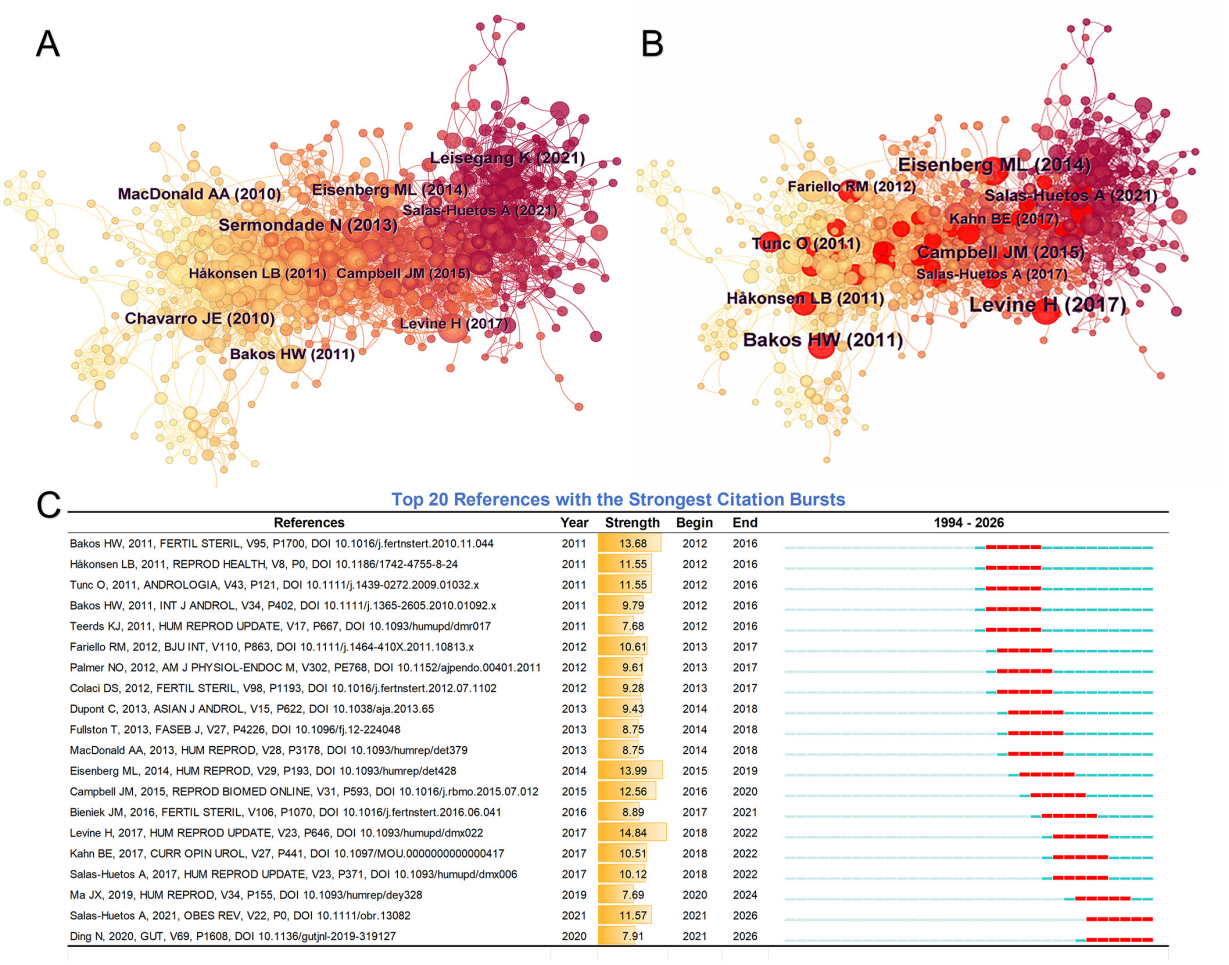
**(A)** Co-citations references network. Node size indicates frequency, and color indicates different years. **(B)** Top 10 Bursts of co-citations references. Nodes in red indicate burst literature. **(C)** Top 20 burst citations references chart.

To explore the evolution of knowledge pathways and the core literature in this field, a co-citation network was constructed using CiteSpace. Default parameter settings were maintained, resulting in a network comprising 858 nodes and 3,917 connections, as shown in Figure 7A. Based on node size, two primary temporal clusters emerged: an early core literature cluster represented by yellow/orange nodes, with prominent nodes including Sermondade N (2013), MacDonald AA (2010), Chavarro JE (2010), Bakos HW (2011), Håkonsen LB (2011), Eisenberg ML (2014), and Campbell JM (2015). These publications form the field's foundational knowledge base. The recent research cluster, represented by deep red and purple nodes, includes core nodes such as Levine H (2017), Leisegang K (2021), and Salas-Huetos A (2021). These nodes exhibit darker colors and denser connections, indicating exceptionally high co-citation frequencies in recent years.

#### 3.7.2 Citation burst analysis

To identify shifts in research hotspots and landmark achievements across different periods in this field, CiteSpace was employed to detect citation bursts. Figure 7B highlights the top 10 references with citation bursts, and Figure 7C details the top 20 burst references ranked by burst intensity and duration, respectively. Levine H (2017) ranked first with an intensity of 14.84, indicating that research on “declining Sperm counts in Western men” has garnered significant academic attention. Subsequently, Eisenberg ML (2014) (Strength = 13.99) and Bakos HW (2011) (Strength = 13.68) represent peaks in male health-related studies and early mechanism research, respectively.

From the perspective of burst time periods, these 20 publications are clearly divided into three phases: 2011–2017 (Initial Burst Phase), which includes Bakos HW (2011), Tunc O (2011), and Fullston T (2013). The emergence period for these papers primarily spanned 2012-2016, marking a critical phase for establishing foundational theories in this field. 2017–2019 (Intermediate Transition Phase): Represented by Levine H (2017) and Salas-Huetos A (2017), with emergence periods primarily spanning 2018–2022. 2020–2026 (Current Sustained Emergence Period): Salas-Huetos A (2021) and Ding N (2020) represent ongoing emergence (End Year = 2026), indicating dietary interventions and specific lifestyle factors as the most active research frontiers today.

#### 3.7.3 Co-citation reference clustering analysis

**Figure 8:**
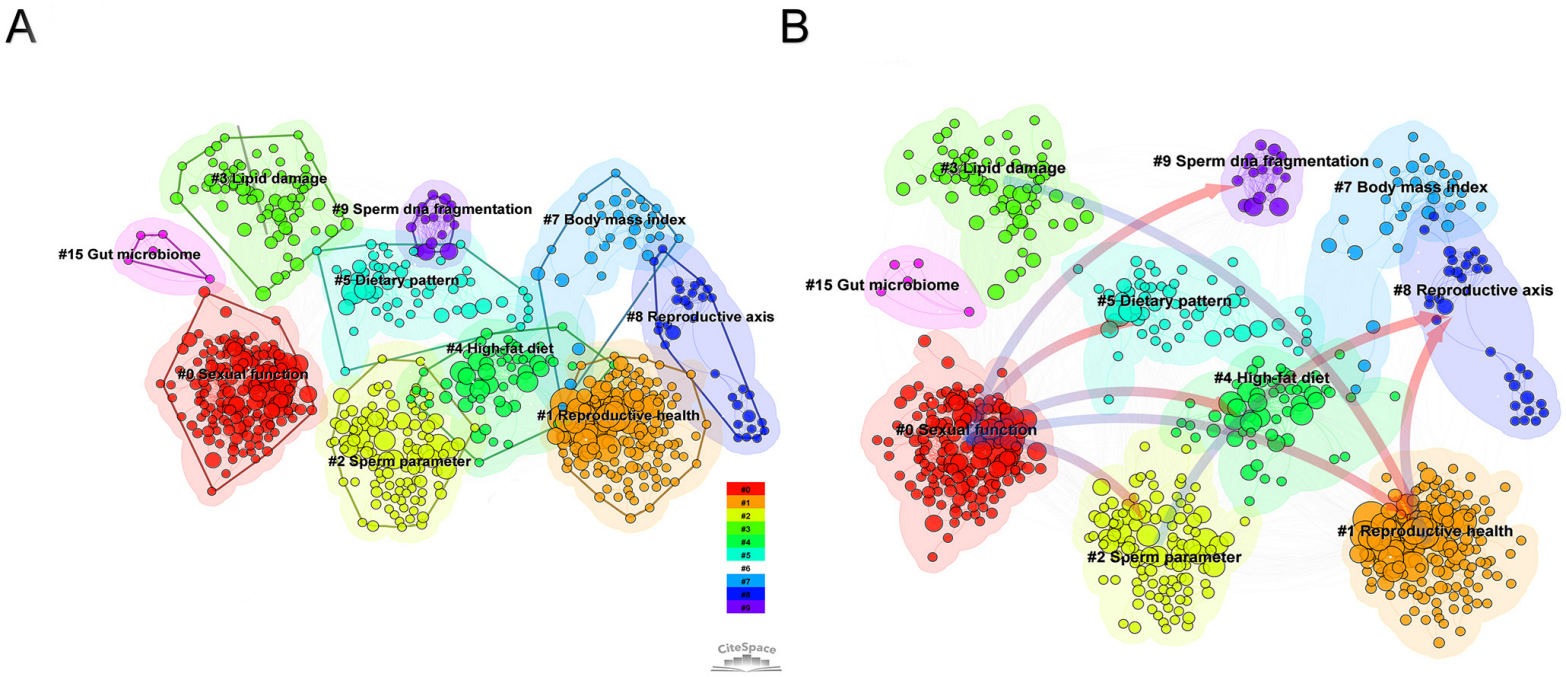
**(A)** Clustered network of co-cited references. **(B**) Cluster dependency and knowledge flow map. Arrows indicate the direction of citation influence, revealing logical dependencies between research themes.

To further reveal the knowledge base structure and major research branches in this field, a clustering analysis of the co-cited network was performed using the log-likelihood algorithm, as shown in Figure 8A. The clustering results yielded a Modularity Q value of 0.6708 (>0.3) and a Weighted Mean Silhouette S value of 0.8463 (>0.7). Both key metrics reached extremely high levels, indicating highly significant cluster structures with exceptional internal homogeneity and robust reliability.

The results identified 15 distinct clusters: #0 Sexual function, #1 Reproductive health, #2 Sperm parameter, #7 Body mass index, #3 Lipid damage, #8 Reproductive axis, #9 Sperm DNA fragmentation, #4 High-fat diet, #5 Dietary pattern, and #15 Gut microbiome.

#### 3.7.4 Co-citation reference cluster dependency analysis

To elucidate the directions of knowledge flow and the logical dependencies among different research themes, CiteSpace generated a cluster dependency view, as shown in Figure 8B. Two distinct knowledge flow hubs are identifiable:

Clinical impact flow originating from #0 Sexual function: Cluster #0, the largest node group, projects multiple significant outward arrows pointing to #5 Dietary pattern, #9 Sperm DNA fragmentation (via intermediate pathways), and #4 High-fat diet. This indicates that clinical observations regarding sexual function and obesity form a crucial foundation for subsequent research on dietary patterns and microscopic Sperm damage.

The mechanism and intervention stream anchored by #4 High-fat diet: Cluster #4 occupies a central position in the network, serving as a pivotal hub connecting the clinical and mechanistic domains. It receives connections from #0 while projecting strong arrows toward #8 (Reproductive axis) and #1 (Reproductive health). This highlights the “high-fat diet model” as a pivotal bridge in explaining reproductive axis disruption and overall reproductive health. Furthermore, #5 Dietary pattern and #9 Sperm DNA fragmentation are positioned at the downstream end of the flow, indicating they represent current specific research endpoints in this field.

## 4 Discussion

### 4.1 Global research trends and knowledge framework evolution in obesity/male infertility

Our bibliometric analysis identifies 2012 as a pivotal inflection point in research on obesity and sperm parameters. Following a period of gradual accumulation (1994–2011, with most years producing 0–5 papers), publication volume surged from 20 papers in 2012 to 36 in 2013, then sustained growth, reaching a peak of 126 in 2021 (Figure 2A). As illustrated by the keyword burst analysis (Figure 6B), foundational terms such as “Body Mass Index,” “Semen Quality,” and “Male Infertility” simultaneously exhibited strong citation bursts beginning in 2012. This trajectory coincides with the publication of several landmark studies (13–15). Thus, the sharp increase in publication volume after 2012 is not merely quantitative but reflects a thematic shift toward molecular mechanisms, as captured by our keyword burst analysis.

Countries/regions analysis reveals a dissociation between publication quantity and impact. While China leads in total publication volume, the United States dominates in citation impact, H-index, and centrality within the collaboration network, highlighting a discrepancy between output quantity and influence. The United States, the United Kingdom, and Italy serve as global hubs for knowledge dissemination, facilitating extensive cross-border collaboration. Conversely, European nations such as Denmark, Sweden, and Belgium exhibit a pattern of “high quality over quantity,” likely due to their well-established reproductive health cohorts and long-term accumulation of epidemiological data. Regarding funding (Table 2), investment is concentrated in a few high-intensity regions, with the NIH and NSFC leading. This distribution mirrors publication trends, underscoring that sustained, systematic public funding is a prerequisite for driving high-impact research in male reproductive health.

Institutional analysis reveals a multipolar collaboration network. Although Harvard University leads in publication volume, the University of Copenhagen and the University of Adelaide demonstrate superior academic influence and network centrality. As shown in Table 3, both institutions achieved an H-index of 21, with citation counts of 2,252 and 1,947, respectively. Figure 4A further shows that the University of Adelaide (TLS = 29) and the University of Copenhagen (TLS = 29) serve as the two core hubs within the collaboration network, surpassing Harvard University (TLS = 14) in total link strength. This suggests that while Harvard dominates in scale, Copenhagen and Adelaide excel in research quality and global connectivity. Network analysis (Figure 4A) further delineates a decentralized collaboration model: an Australian cluster centered on the University of Adelaide exhibits strong regional cohesion, whereas a Euro-American cluster led by Harvard, Copenhagen, and the Cleveland Clinic drives frontier exploration through intensive transcontinental collaboration. This structure, balancing regional density with global breadth, effectively accelerates knowledge dissemination in reproductive medicine.

A blend of specialized focus and broad impact characterizes journal distribution. According to Table 4, ANDROLOGIA and ANDROLOGY served as the primary venues for publication volume. However, in terms of academic influence, FERTILITY AND STERILITY and Human Reproduction dominated. As shown in Table 4, most of the top ten journals by publication volume are in the Q1 quartile, while Asian Journal of Andrology and PLOS ONE are in Q2, and ANDROLOGIA is in Q3. These Q1 journals not only represent the highest academic standards but also reflect how research on obesity-related male infertility has transcended niche boundaries to become a central focus in mainstream reproductive medicine.

Dual-map overlay analysis (Figure 4C) illuminates the field's evolutionary trajectory, highlighting a transition from “clinical phenomena to molecular mechanisms.” The primary citation pathways extend from the “Medicine, Medical, Clinical” cluster (left) to the “Molecular, Biology, Genetics” cluster (right). A secondary yellow pathway from “Molecular, Biology, Immunology” to “Molecular, Biology, Genetics” (z = 3.83, f = 2,684) indicates additional cross-disciplinary influence. This pattern illustrates a clear shift in research focus: clinical observations are increasingly being investigated at the molecular and genetic levels to uncover underlying pathologies. Such interdisciplinary knowledge flow not only deepens our understanding of mechanisms but also provides a theoretical basis for developing precision interventions.

The author's analysis indicates that Jorge E. Chavarro and Michael L. Eisenberg are tied for the lead with 16 publications each. Among them, Chavarro tops both the H-index and total citation counts, solidifying his academic standing in the field. Timeline analysis (Figure 5C) reveals that Chavarro pioneered demonstrating the effects of dietary and lifestyle factors on male fertility through large-scale epidemiological cohort studies between 2012 and 2016 (16, 17). Within the author collaboration network, the Italian research team centered around Salonia Andrea, Montorsi Francesco, and Boeri Luca, despite having a slightly lower publication volume, exhibits exceptionally high total link strength. This reflects the intensity of their internal collaboration and positions them as leaders in European multicenter clinical research.

### 4.2 Research hotspots and future trends in the field of obesity/obesity/male infertility

As visualized in the CiteSpace clustering network (Figure 8A) and keyword co-occurrence map (Figure 6A), the diverse research themes can be systematically categorized. The cluster dependency network (Figure 8B) further maps the conceptual flow among these themes. Based on keyword co-occurrence, CiteSpace reference highlighting, and the latest clustering dependency network, the key research areas in this field can be summarized as: oxidative stress-driven sperm DNA damage (Clusters #3 and #9); the gut-testicular axis and metabolic immune regulation (Cluster #15); multidimensional intervention approaches (Clusters #4 and #5); and the epigenetics and transgenerational effects of paternal obesity.

#### 4.2.1 Oxidative stress-driven sperm DNA damage

Research in male infertility is pivoting from macroscopic semen analysis to the assessment of molecular integrity. Our bibliometric findings strongly support this clinical transition. As shown in Figure 8A, “Lipid damage” (Cluster #3) and “sperm DNA fragmentation” (Cluster #9) have emerged as highly concentrated research hubs. Furthermore, the keyword burst analysis (Figure 6B) highlights that “sperm DNA” became a major research focus starting in 2021.

This bibliometric trend is strongly supported by recent biological consensus: obesity-induced systemic metabolic dysfunction is the primary driver of molecular sperm damage, with oxidative stress acting as the central pathological mediator. While physiological levels of reactive oxygen species (ROS) are essential (18), chronic inflammation associated with obesity disrupts redox homeostasis (19). This explains the prominence of Cluster #3 (Lipid damage): the sperm plasma membrane is uniquely vulnerable to lipid peroxidation, which generates genotoxic aldehydes (20). The emergence of “Sperm DNA” as a recent keyword burst (Figure 6B) reflects the clinical realization that exogenous oxidative stress induces intracellular Ca^2+^ overload and ATP depletion, directly leading to DNA fragmentation (21).

Crucially, this subclinical molecular damage explains a common clinical paradox: many obese men with normozoospermia (normal sperm count and motility) suffer from recurrent pregnancy loss (RPL) or unexplained infertility. Clinical data confirm that the sperm DNA fragmentation (SDF) index has emerged as an independent predictor of RPL (22, 23) and poor assisted reproductive technology (ART) outcomes (24). These findings underscore that even morphologically normal sperm, if burdened with obesity-induced oxidative damage, possess severely compromised post-fertilization developmental potential.

Given this mechanism, the clinical translation of SDF testing has become a focal point. Future diagnostic strategies must move beyond traditional parameters to integrate SDF into routine fertility assessments for obese men. Concurrently, antioxidant interventions targeting ROS pathways offer a promising avenue to restore genomic integrity at its source, thereby improving pregnancy outcomes and safeguarding offspring health.

#### 4.2.2 The gut-testicular axis and metabolic immune regulation

A striking feature of the recent knowledge map is the emergence of the “Gut microbiome” (Cluster #15, Figure 8A). The cluster dependency network (Figure 8B) illustrates directional links between this cluster and dietary factors, suggesting a systemic research approach. Additionally, the highly burst reference by Ding N (25) confirmed that high-fat diet (HFD)-induced dysbiosis impairs spermatogenesis. Building on this foundation, recent fecal microbiota transplantation experiments demonstrated that transferring microbiota from obese to germ-free mice recapitulates testicular inflammation and reduced sperm quality in recipients (26). These findings provide compelling causal evidence for the gut microbiome's role in male reproduction.

This systemic damage is inextricably linked to poor dietary patterns. Long-term HFD consumption remodels the gut microbiome, characterized by an altered Firmicutes/Bacteroidetes ratio and reduced probiotic abundance (27). This dysbiosis triggers the translocation of pro-inflammatory factors, such as bacterial lipopolysaccharides, into circulation, creating a state of metabolic endotoxemia. Circulating endotoxins activate TLR4/NF-κB signaling in Leydig and Sertoli cells, inducing local inflammation that compromises the blood-testis barrier and exposes immune-privileged germ cells to systemic attack (28). Beyond direct immune injury, the gut microbiota modulates the testicular microenvironment via metabolites. Dysbiosis significantly reduces beneficial short-chain fatty acids (SCFAs), which are crucial for maintaining lipid homeostasis and antioxidant defenses in the testes (26, 29). The resulting accumulation of oxidative stress leads to mitochondrial dysfunction and DNA fragmentation (Cluster #9), impairing fertilization capacity (30).

The identification of the “gut-testis axis” offers a novel therapeutic entry point. Since reproductive damage originates in the gut, restoring microbial balance represents a viable strategy. Targeted nutritional interventions (31) and probiotics (32) have shown efficacy in repairing the intestinal barrier, reducing endotoxemia, and alleviating testicular oxidative stress. Future research should focus on blocking this inflammatory pathway to provide obese men with a non-hormonal, biologically-based treatment option.

#### 4.2.3 Epigenetics of paternal obesity and transgenerational effects

The most forward-looking insight from our keyword analysis is the sudden and powerful citation burst of the term “Family” in 2025-2026 (Figure 6B). This transition from “male infertility” (bursting in 2012) to “Family” signifies that the scope of reproductive medicine is expanding from treating individual infertility to addressing the intergenerational implications of family health, aligning perfectly with the “Paternal Origins of Health and Disease” hypothesis (33, 34).

Underpinning this emerging keyword trend is growing evidence that paternal environmental exposures alter germ cell epigenetics. At the RNA level, sperm small non-coding RNAs serve as key carriers of metabolic information (35, 36). Furthermore, paternal obesity exerts enduring effects through histone modifications and DNA methylation, causing severe metabolic dysfunction in offspring (37). Notably, human cohort studies, such as those from the PACE consortium, reveal significant correlations between paternal preconception BMI and DNA methylation at specific loci in neonatal cord blood (38, 39). Recent data suggest these effects can even transcend generations (transgenerational inheritance), with paternal obesity affecting gene expression in the F2 generation's liver via germline methylation memory (40, 41).

Unlike genetic mutations, epigenetic modifications are plastic, offering a critical window for intervention. Studies show that in HFD-induced obesity models, moderate caloric restriction or exercise can reverse abnormal epigenetic marks in sperm by activating the AMPK/SIRT1 pathway, effectively blocking the transmission of metabolic disease (42). This growing realization that paternal interventions can directly safeguard offspring's health perfectly explains the sudden surge of the keyword “Family” in our bibliometric landscape (Figure 6B). It indicates that incorporating “paternal preconception health optimization” into clinical guidelines to interrupt the transmission of metabolic disease is becoming the new frontier in this field.

#### 4.2.4 Multidimensional intervention approaches

As the mechanisms of damage diversify, the intervention strategies mapped in our network have correspondingly evolved. The co-occurrence network (Figure 6A) features prominent nodes, including “weight loss,” “bariatric surgery,” and “*in vitro* fertilization.” Moreover, “Oral Antioxidant Treatment” exhibited a distinct keyword burst starting in 2020 (Figure 6B), and “Dietary pattern” forms a distinct cluster (Cluster #5, Figure 8A). These metrics reflect a shift from monotherapy to multimodal management. This diversification in research is driven by a complex clinical reality: intervention efficacy varies significantly, and improvements in sperm quality are not strictly proportional to weight loss. In fact, as highlighted by recent literature, some aggressive interventions may even carry acute reproductive risks (43).

However, efficacy varies, and improvements in sperm quality are not linearly proportional to weight loss; some interventions may even carry risks (43).

**Dietary Interventions**: Highly co-cited burst papers, such as Salas-Huetos A (2017 & 2021, Figure 7C), have driven the research focus from simple caloric restriction to comprehensive nutritional restructuring. A prime example is the Mediterranean diet, rich in antioxidants and unsaturated fatty acids, which has garnered attention for its multi-target benefits (44); it not only modulates the gut microbiota to reduce systemic inflammation but also provides methyl donors essential for sperm DNA methylation homeostasis. Furthermore, this nutritional shift closely aligns with the 2020 keyword burst in “Oral Antioxidant Treatment” (Figure 6B). Within this context, Omega-3 fatty acid supplementation remains a promising avenue for mitigating oxidative stress and enhancing mitochondrial function (45). Notably, however, while such micronutrients are routinely prescribed, a randomized trial by Schisterman et al. found that zinc and folic acid supplementation alone did not significantly improve fertility outcomes (46). This strongly suggests that therapeutic efficacy depends on specific metabolic contexts and overall dietary patterns, rather than isolated micronutrient supplementation.

**Exercise:** As explicitly mapped in our network analysis, exercise interventions act as a critical bridge between “Body mass index” (Cluster #7) and the “Reproductive axis” (Cluster #8). Regular physical activity is an independent protective factor. Moderate-intensity aerobic exercise enhances semen quality by reducing visceral fat (47) and restoring the hypothalamic-pituitary-gonadal (HPG) axis (48, 49). Mechanistically, exercise induces skeletal muscle secretion of myokines (e.g., irisin), which cross-talk with testicular tissue to upregulate mitochondrial biogenesis PGC-1α pathway (50) and antioxidant enzymes (51). However, the “principle of moderation” is critical; high-intensity or exhaustive exercise may induce acute oxidative stress, exacerbating DNA damage (52). Thus, to maximize reproductive benefits without triggering molecular injury, exercise prescriptions for obese men must be carefully individualized and progressive.

**Bariatric Surgery:** As a prominent network node (Figure 6A), bariatric surgery offers rapid metabolic remodeling and reversal of hypogonadotropic hypogonadism for severe obesity. This significant recovery of testosterone and SHBG levels directly aligns with the “Reproductive axis” (Cluster #8) (53, 54). However, its impact on semen parameters is paradoxical. While hormonal profiles improve, sperm quality may not follow suit, and temporary deterioration is common in the early postoperative period (< 12 months) (55, 56). Mechanisms include rapid weight-loss-induced oxidative stress, nutritional malabsorption (57), and the release of lipophilic endocrine-disrupting chemicals (EDCs) from adipose tissue stores (58). Additionally, surgery-induced shifts in the gut microbiome **(Cluster #15)** may influence spermatogenesis via the “gut-brain-testis axis” (59). Therefore, perioperative nutritional monitoring and toxin assessment are vital and require a multidisciplinary approach.

Interventions for obese men must extend beyond treating infertility to interrupting the intergenerational cycle of metabolic disease. We propose the concept of a “Paternal Preconception Health Window.” During this critical period, integrating optimized nutrition, exercise, and antioxidant therapy can enhance reproductive potential and reshape sperm epigenetic information, providing offspring with an optimal developmental foundation. Future research should prioritize precision interventions based on gut microbiota and epigenetic markers to transition from “treating infertility” to “building family health.”

### 4.3 Limitations

This study has several limitations. First, only Web of Science and Scopus were used; other databases (e.g., PubMed, Google Scholar) were excluded, which may have introduced database selection bias. Second, only English-language articles were included, potentially overlooking relevant non-English studies. Third, bibliometric methods inherently rely on citation-based indicators that reflect academic impact rather than direct scientific quality, and the results are sensitive to analytical parameter choices. Additionally, the analysis is based on 1,222 papers, which may not fully capture emerging research directions. Future studies should incorporate more databases, include multilingual publications, and complement bibliometric analysis with qualitative reviews.

## 5 Conclusion

This study comprehensively maps the global research landscape of obesity and male infertility, tracing the field's evolution and identifying emerging frontiers. These insights provide a roadmap for future research, offering a deeper understanding of the field and advancing the development of precision reproductive medicine.

## Data Availability

The original contributions presented in the study are included in the article/Supplementary material, further inquiries can be directed to the corresponding authors.
