# Supplementary material for: Global research trends on the impact of obesity on male infertility: a bibliometric analysis
Source: Front Nutr. 2026 May 11;13:1817082. doi: 10.3389/fnut.2026.1817082 (PMC13199085; doi:10.3389/fnut.2026.1817082)
Supplement: Supplementary file 1 [file Table_1.docx]

Supplementary Material

# Supplementary Data

- 1. Detailed Data of the National Cooperation Map （Figure 3A）

| **id** | **label** | **x** | **y** | **cluster** | **weight<Links>** | **weight<Total link strength>** | **weight<Documents>** | **weight<Citations>** | **weight<Norm. citations>** | **score<Avg. pub. year>** | **score<Avg. citations>** | **score<Avg. norm. citations>** |
| --- | --- | --- | --- | --- | --- | --- | --- | --- | --- | --- | --- | --- |
| 1 | Argentina | 0.5712 | -0.215 | 5 | 12 | 14 | 13 | 768 | 26.5531 | 2019.2308 | 59.0769 | 2.0425 |
| 2 | Australia | 0.4056 | 0.4467 | 4 | 19 | 34 | 56 | 3389 | 98.8066 | 2018.5357 | 60.5179 | 1.7644 |
| 3 | Austria | -0.8718 | 0.3297 | 1 | 4 | 5 | 5 | 107 | 1.7527 | 2016.8 | 21.4 | 0.3505 |
| 4 | Belgium | -0.6869 | 0.5203 | 1 | 10 | 14 | 12 | 871 | 26.7175 | 2019 | 72.5833 | 2.2265 |
| 6 | Brazil | 0.015 | -0.2102 | 6 | 21 | 46 | 43 | 2228 | 80.3275 | 2018.4884 | 51.814 | 1.8681 |
| 8 | Canada | -0.0931 | 0.5256 | 6 | 4 | 8 | 18 | 756 | 25.232 | 2018.6667 | 42 | 1.4018 |
| 9 | Chile | -0.6571 | 0.5835 | 1 | 10 | 11 | 6 | 388 | 13.3855 | 2021.6667 | 64.6667 | 2.2309 |
| 10 | China | -0.2348 | -0.1441 | 6 | 20 | 50 | 203 | 4982 | 254.8892 | 2020.9507 | 24.5419 | 1.2556 |
| 11 | Croatia | -0.8185 | 0.6231 | 1 | 5 | 6 | 7 | 91 | 6.3982 | 2021.2857 | 13 | 0.914 |
| 13 | Czech Republic | 1.3518 | -0.2787 | 5 | 1 | 1 | 6 | 87 | 3.4846 | 2018.8333 | 14.5 | 0.5808 |
| 15 | Denmark | 0.4918 | 0.0446 | 3 | 18 | 58 | 43 | 2858 | 75.3028 | 2017.9535 | 66.4651 | 1.7512 |
| 17 | Egypt | -0.244 | -0.671 | 7 | 13 | 18 | 17 | 522 | 27.9471 | 2020.2353 | 30.7059 | 1.6439 |
| 20 | France | 1.1493 | -0.2334 | 5 | 12 | 20 | 39 | 1679 | 40.3368 | 2018.6923 | 43.0513 | 1.0343 |
| 21 | Germany | -0.3689 | 0.1686 | 1 | 14 | 19 | 26 | 929 | 31.6428 | 2018.8462 | 35.7308 | 1.217 |
| 23 | Greece | 0.0561 | 0.7815 | 4 | 4 | 9 | 16 | 677 | 37.8255 | 2020.1875 | 42.3125 | 2.3641 |
| 25 | Hungary | 0.7019 | -0.2541 | 5 | 10 | 11 | 5 | 509 | 9.1248 | 2018.4 | 101.8 | 1.825 |
| 27 | India | -0.5742 | -0.7342 | 2 | 10 | 17 | 19 | 628 | 25.5242 | 2019.8947 | 33.0526 | 1.3434 |
| 29 | Iran | -0.0798 | -0.0031 | 1 | 7 | 12 | 39 | 846 | 50.415 | 2020.641 | 21.6923 | 1.2927 |
| 33 | Italy | 0.3828 | -0.1553 | 3 | 25 | 60 | 91 | 3478 | 159.0411 | 2019.8901 | 38.2198 | 1.7477 |
| 34 | Japan | 0.1305 | 0.8251 | 4 | 3 | 5 | 20 | 312 | 18.8294 | 2020.55 | 15.6 | 0.9415 |
| 39 | Malaysia | -0.6501 | -0.9244 | 2 | 12 | 17 | 15 | 673 | 38.9074 | 2021.6667 | 44.8667 | 2.5938 |
| 41 | Mexico | 0.846 | -0.391 | 8 | 3 | 3 | 7 | 124 | 9.768 | 2021.8571 | 17.7143 | 1.3954 |
| 43 | Netherlands | 0.7081 | 0.0843 | 3 | 14 | 26 | 18 | 795 | 25.3138 | 2020.3889 | 44.1667 | 1.4063 |
| 44 | New Zealand | 0.5899 | 0.6812 | 4 | 1 | 1 | 5 | 245 | 7.7752 | 2018.6 | 49 | 1.555 |
| 45 | Nigeria | -0.7575 | -1.0853 | 2 | 2 | 3 | 9 | 135 | 10.8928 | 2021.4444 | 15 | 1.2103 |
| 46 | Norway | 0.6907 | 0.3736 | 3 | 7 | 9 | 7 | 360 | 7.6174 | 2016.7143 | 51.4286 | 1.0882 |
| 47 | Pakistan | 0.0142 | -0.6276 | 7 | 3 | 8 | 9 | 155 | 6.0138 | 2018.5556 | 17.2222 | 0.6682 |
| 48 | Poland | 0.7914 | 0.2578 | 3 | 6 | 11 | 22 | 501 | 22.9652 | 2019.7727 | 22.7727 | 1.0439 |
| 49 | Portugal | -0.5154 | -0.1903 | 2 | 14 | 26 | 22 | 1217 | 49.5034 | 2019.8182 | 55.3182 | 2.2502 |
| 50 | Qatar | -0.4669 | -0.5614 | 2 | 12 | 19 | 5 | 422 | 16.5366 | 2018.2 | 84.4 | 3.3073 |
| 52 | Russia | -0.8294 | 0.6521 | 1 | 3 | 4 | 5 | 50 | 3.8273 | 2020.8 | 10 | 0.7655 |
| 53 | Saudi Arabia | 0.1324 | -0.5689 | 7 | 14 | 23 | 15 | 705 | 18.5806 | 2019.1333 | 47 | 1.2387 |
| 58 | South Africa | -0.4548 | -0.6751 | 2 | 13 | 21 | 14 | 1191 | 57.2797 | 2019.6429 | 85.0714 | 4.0914 |
| 59 | South Korea | -0.9044 | 0.2973 | 1 | 3 | 3 | 7 | 47 | 1.3739 | 2018.5714 | 6.7143 | 0.1963 |
| 60 | Spain | 0.5616 | -0.1222 | 8 | 13 | 35 | 47 | 2079 | 79.9814 | 2019.0851 | 44.234 | 1.7017 |
| 62 | Sweden | 0.2383 | 0.4108 | 3 | 17 | 37 | 23 | 2169 | 77.8513 | 2018.4783 | 94.3043 | 3.3848 |
| 66 | Turkey | -0.3227 | 0.2579 | 1 | 6 | 6 | 34 | 458 | 14.2577 | 2019.0588 | 13.4706 | 0.4193 |
| 69 | United Kingdom | -0.1971 | 0.3401 | 4 | 23 | 49 | 45 | 1808 | 81.6676 | 2020.2444 | 40.1778 | 1.8148 |
| 70 | United States | -0.1008 | -0.1585 | 6 | 28 | 141 | 180 | 9074 | 325.5337 | 2018.9667 | 50.4111 | 1.8085 |

- 1. Detailed Data of the Institution Cooperation Map （Figure 4A）

| **id** | **label** | **x** | **y** | **cluster** | **weight<Links>** | **weight<Total link strength>** | **weight<Documents>** | **weight<Citations>** | **weight<Norm. citations>** | **score<Avg. pub. year>** | **score<Avg. citations>** | **score<Avg. norm. citations>** |
| --- | --- | --- | --- | --- | --- | --- | --- | --- | --- | --- | --- | --- |
| 2 | Aarhus University | -0.1007 | -0.0019 | 2 | 6 | 12 | 14 | 540 | 16.9907 | 2018.7857 | 38.5714 | 1.2136 |
| 53 | Androfert | -0.1443 | -0.0017 | 2 | 6 | 10 | 5 | 593 | 35.769 | 2020.6 | 118.6 | 7.1538 |
| 208 | Cleveland Clinic | -0.5458 | -0.0158 | 1 | 8 | 13 | 14 | 1439 | 57.5688 | 2020.2857 | 102.7857 | 4.1121 |
| 436 | Harvard University | 0.5159 | -0.0093 | 5 | 4 | 14 | 32 | 1215 | 30.4828 | 2018.0938 | 37.9688 | 0.9526 |
| 536 | Imperial College London | -0.8152 | -0.0144 | 1 | 5 | 7 | 7 | 435 | 25.9029 | 2021.1429 | 62.1429 | 3.7004 |
| 647 | Karolinska Institute | 0.2262 | 0.0016 | 2 | 2 | 3 | 10 | 1122 | 28.4693 | 2018.9 | 112.2 | 2.8469 |
| 722 | Lund University | 0.0099 | 0.0028 | 2 | 6 | 8 | 8 | 503 | 30.0697 | 2018.625 | 62.875 | 3.7587 |
| 784 | Monash University | 1.4698 | -0.0025 | 3 | 6 | 20 | 13 | 511 | 15.9586 | 2018.4615 | 39.3077 | 1.2276 |
| 960 | Repromed | 1.582 | -0.0027 | 3 | 2 | 15 | 13 | 711 | 19.5538 | 2018.6923 | 54.6923 | 1.5041 |
| 995 | Sapienza University Of Rome | -0.6078 | 0.026 | 1 | 4 | 5 | 8 | 240 | 17.2022 | 2020.75 | 30 | 2.1503 |
| 1073 | Stanford University | -0.1744 | 0.0136 | 2 | 7 | 11 | 18 | 893 | 41.4293 | 2019.8333 | 49.6111 | 2.3016 |
| 1431 | University College London | -1.0705 | -0.0274 | 4 | 4 | 7 | 6 | 207 | 8.6194 | 2019.1667 | 34.5 | 1.4366 |
| 1432 | University Of Adelaide | 1.4873 | -0.0025 | 3 | 6 | 29 | 24 | 1497 | 37.2074 | 2017.4583 | 62.375 | 1.5503 |
| 1433 | University Of Aveiro | -1.1276 | -0.0305 | 4 | 3 | 10 | 9 | 235 | 19.353 | 2021.1111 | 26.1111 | 2.1503 |
| 1434 | University Of Beira Interior | -1.1185 | -0.0299 | 4 | 3 | 9 | 6 | 335 | 8.1949 | 2017.1667 | 55.8333 | 1.3658 |
| 1435 | University Of Campinas | -0.1701 | -0.0038 | 2 | 4 | 6 | 6 | 395 | 9.4619 | 2015 | 65.8333 | 1.577 |
| 1438 | University Of Copenhagen | 0.5657 | -0.0011 | 5 | 12 | 29 | 23 | 1693 | 42.8651 | 2017.2609 | 73.6087 | 1.8637 |
| 1442 | University Of Melbourne | 1.3649 | -0.0023 | 3 | 5 | 8 | 8 | 463 | 7.9492 | 2015.75 | 57.875 | 0.9937 |
| 1443 | University Of Miami | -0.7319 | 0.0024 | 1 | 6 | 7 | 6 | 279 | 16.4952 | 2021.8333 | 46.5 | 2.7492 |
| 1444 | University Of Milan | -0.6269 | 0.057 | 1 | 2 | 7 | 7 | 103 | 9.0514 | 2021.7143 | 14.7143 | 1.2931 |
| 1445 | University Of Murcia | 0.5931 | -0.0086 | 5 | 3 | 12 | 5 | 411 | 6.6943 | 2013.6 | 82.2 | 1.3389 |
| 1446 | University Of Naples Federico Ii | -0.6239 | 0.0468 | 1 | 3 | 6 | 6 | 116 | 11.4403 | 2022.1667 | 19.3333 | 1.9067 |
| 1449 | University Of Porto | -1.0461 | -0.0269 | 4 | 7 | 22 | 15 | 822 | 38.625 | 2019.4 | 54.8 | 2.575 |
| 1450 | University Of Rochester | 0.5892 | -0.0098 | 5 | 3 | 11 | 6 | 381 | 6.5934 | 2015.5 | 63.5 | 1.0989 |
| 1452 | University Of Sydney | 1.2771 | -0.0022 | 3 | 5 | 10 | 6 | 249 | 13.5632 | 2019.3333 | 41.5 | 2.2605 |
| 1453 | University Of Tehran | -0.671 | 0.0164 | 1 | 2 | 2 | 16 | 333 | 17.6548 | 2020.4375 | 20.8125 | 1.1034 |
| 1454 | University Of Utah | -0.1156 | 0.0018 | 2 | 4 | 4 | 15 | 1156 | 64.8005 | 2018.8 | 77.0667 | 4.32 |
| 1455 | University Of Western Australia | 1.3249 | -0.0022 | 3 | 5 | 11 | 7 | 157 | 6.5811 | 2019.4286 | 22.4286 | 0.9402 |
| 1456 | University Of Western Cape | -0.7162 | -0.0181 | 1 | 4 | 9 | 12 | 1138 | 55.5659 | 2019.6667 | 94.8333 | 4.6305 |
| 1484 | Vita-Salute San Raffaele University | -0.5995 | 0.0452 | 1 | 5 | 15 | 16 | 367 | 32.4101 | 2021.1875 | 22.9375 | 2.0256 |

- 1. Detailed Data of the Author Cooperation Map （Figure 5A）

| **id** | **label** | **x** | **y** | **cluster** | **weight<Links>** | **weight<Total link strength>** | **weight<Documents>** | **weight<Citations>** | **weight<Norm. citations>** | **score<Avg. pub. year>** | **score<Avg. citations>** | **score<Avg. norm. citations>** |
| --- | --- | --- | --- | --- | --- | --- | --- | --- | --- | --- | --- | --- |
| 56 | Agarwal, Ashok | 0.9779 | -0.2421 | 3 | 5 | 12 | 10 | 1271 | 47.9405 | 2019.7 | 127.1 | 4.7941 |
| 117 | Alfano, Massimo | 0.0705 | 0.4467 | 1 | 10 | 43 | 5 | 86 | 5.4496 | 2021.4 | 17.2 | 1.0899 |
| 155 | Alves, Marco G. | 1.1633 | -0.2188 | 3 | 7 | 23 | 14 | 742 | 35.4094 | 2019.4286 | 53 | 2.5292 |
| 413 | Belladelli, Federico | -0.2444 | 0.161 | 1 | 11 | 58 | 8 | 121 | 9.7106 | 2022.375 | 15.125 | 1.2138 |
| 509 | Boeri, Luca | -0.2753 | 0.3286 | 1 | 11 | 73 | 10 | 163 | 9.6528 | 2021.2 | 16.3 | 0.9653 |
| 662 | Candela, Luigi | 0.1115 | 0.3058 | 1 | 10 | 43 | 5 | 63 | 5.6713 | 2022.2 | 12.6 | 1.1343 |
| 680 | Capogrosso, Paolo | -0.0581 | 0.3879 | 1 | 10 | 61 | 9 | 167 | 9.0672 | 2020.6667 | 18.5556 | 1.0075 |
| 702 | Carrell, Douglas T. | -0.6619 | -0.0886 | 2 | 2 | 6 | 9 | 766 | 28.3955 | 2017.1111 | 85.1111 | 3.1551 |
| 729 | Cazzaniga, Walter | -0.0676 | 0.5312 | 1 | 10 | 39 | 5 | 103 | 4.6696 | 2019.8 | 20.6 | 0.9339 |
| 771 | Chavarro, Jorge E. | -1.0049 | -0.287 | 2 | 6 | 31 | 12 | 579 | 15.2662 | 2018.25 | 48.25 | 1.2722 |
| 1249 | Durairajanayagam, Damayanthi | 0.9942 | -0.047 | 3 | 6 | 9 | 5 | 298 | 13.3697 | 2020.8 | 59.6 | 2.6739 |
| 1278 | Eisenberg, Michael L. | -0.4656 | 0.0168 | 2 | 6 | 7 | 13 | 730 | 37.7272 | 2020.6923 | 56.1538 | 2.9021 |
| 1375 | Fallara, Giuseppe | -0.0109 | 0.2519 | 1 | 10 | 43 | 5 | 67 | 6.3507 | 2022.6 | 13.4 | 1.2701 |
| 1583 | Gaskins, Audrey J. | -1.1361 | -0.3008 | 2 | 5 | 16 | 7 | 392 | 8.9328 | 2017.4286 | 56 | 1.2761 |
| 1856 | Hauser, Russ | -1.0582 | -0.3737 | 2 | 5 | 19 | 6 | 317 | 6.6368 | 2016.3333 | 52.8333 | 1.1061 |
| 1887 | Henkel, Ralf | 0.9906 | -0.1661 | 3 | 5 | 12 | 7 | 818 | 47.5786 | 2021 | 116.8571 | 6.7969 |
| 1944 | Hotaling, James M. | -0.5973 | -0.0546 | 2 | 2 | 4 | 6 | 623 | 40.1366 | 2019.6667 | 103.8333 | 6.6894 |
| 2501 | Leisegang, Kristian | 0.9076 | -0.2051 | 3 | 5 | 11 | 6 | 729 | 36.2872 | 2019.1667 | 121.5 | 6.0479 |
| 3072 | Minguez-Alarcon, Lidia | -0.9081 | -0.2905 | 2 | 6 | 23 | 6 | 152 | 6.6431 | 2020.6667 | 25.3333 | 1.1072 |
| 3135 | Montorsi, Francesco | -0.1542 | 0.2781 | 1 | 11 | 73 | 10 | 163 | 9.6528 | 2021.2 | 16.3 | 0.9653 |
| 3354 | Oliveira, Pedro F. | 1.2715 | -0.2159 | 3 | 2 | 18 | 13 | 538 | 26.0509 | 2019.3846 | 41.3846 | 2.0039 |
| 3619 | Pozzi, Edoardo | 0.0848 | 0.1337 | 1 | 12 | 65 | 9 | 122 | 11.5663 | 2022.3333 | 13.5556 | 1.2851 |
| 3717 | Ramasamy, Ranjith | 0.8915 | -0.1114 | 3 | 6 | 6 | 6 | 271 | 15.1979 | 2020.6667 | 45.1667 | 2.533 |
| 3909 | Salas-Huetos, Albert | -0.7934 | -0.1565 | 2 | 5 | 13 | 7 | 448 | 25.266 | 2020.8571 | 64 | 3.6094 |
| 3915 | Salonia, Andrea | -0.1023 | 0.1364 | 1 | 11 | 73 | 12 | 214 | 23.9131 | 2021.75 | 17.8333 | 1.9928 |
| 4118 | Silva, Branca M. | 1.261 | -0.3051 | 3 | 2 | 10 | 5 | 255 | 6.0434 | 2017.2 | 51 | 1.2087 |
| 4131 | Singh, Harbindar Jeet | 1.0131 | 0.0181 | 3 | 1 | 4 | 5 | 104 | 4.8585 | 2021.4 | 20.8 | 0.9717 |
| 4196 | Souter, Irene | -0.9429 | -0.1816 | 2 | 6 | 19 | 5 | 82 | 4.4328 | 2021.2 | 16.4 | 0.8866 |
| 4559 | Ventimiglia, Eugenio | -0.1903 | 0.4528 | 1 | 10 | 68 | 9 | 163 | 9.6528 | 2020.7778 | 18.1111 | 1.0725 |
| 4751 | Williams, Paige L. | -1.0659 | -0.2044 | 2 | 6 | 20 | 5 | 154 | 5.1618 | 2019.2 | 30.8 | 1.0324 |
